# The Impact of Artemisinin Combination Therapy and Long-Lasting Insecticidal Nets on Forest Malaria Incidence in Tribal Villages of India, 2006–2011

**DOI:** 10.1371/journal.pone.0056740

**Published:** 2013-02-20

**Authors:** Naman K. Shah, Prajesh Tyagi, Surya K. Sharma

**Affiliations:** National Institute of Malaria Research, New Delhi, India; Johns Hopkins University, United States of America

## Abstract

**Introduction:**

New tools for malaria control, artemisinin combination therapy (ACT) and long-lasting insecticidal nets (LLINs) were recently introduced across India. We estimated the impact of universal coverage of ACT and ACT plus LLINs in a setting of hyperendemic, forest malaria transmission.

**Methods:**

We reviewed data collected through active and passive case detection in a vaccine trial cohort of 2,204 tribal people residing in Sundargarh district, Odisha between 2006 and 2011. We compared measures of transmission at the village and individual level in 2006–2009 versus 2010–2011 after ACT (in all villages) and LLINs (in three villages) were implemented.

**Results:**

During 2006–2009 malaria incidence per village ranged from 156–512 per 1000 persons per year and slide prevalence ranged from 28–53%. Routine indoor residual spray did not prevent seasonal peaks of malaria. Post-intervention impact in 2010–2011 was dramatic with ranges of 14–71 per 1000 persons per year and 6–16% respectively. When adjusted for village, ACT alone decreased the incidence of malaria by 83% (IRR 0.17, 95%CI: 0.10, 0.27) and areas using ACT and LLINs decreased the incidence of malaria by 86% (IRR 0.14, 95%CI: 0.05, 0.38). After intervention, the age of malaria cases, their parasite density, and proportion with fever at the time of screening increased.

**Conclusions:**

ACT, and LLINs along with ACT, effectively reduced malaria incidence in a closely monitored population living in a forest ecotype. It is unclear whether LLINs were impactful when prompt and quality antimalarial treatment was available. In spite of universal coverage, substantial malaria burden remained.

## Introduction

The widespread implementation of artemisinin combination therapy (ACT) for the treatment of *Plasmodium falciparum* and long-lasting insecticidal nets (LLINs) alone or in combination by malaria control programmes have been followed by reports of declines in transmission in several countries [1]–[4]. This is relatively unsurprising given their high efficacy in controlled trials. However, programme conditions may be less rigorous than those maintained in trials and study settings may not generalize over larger geographic areas. Furthermore, trials or impact studies rarely evaluate combinations of interventions, for example, the effect of improved vector control in the context of prompt and effective treatment. Local assessments of anti-malaria impact after the routine adoption of interventions are thus needed. Such assessments of impact have been limited by the lack of routinely collected data available in control programmes.

The control of malaria is a key goal for India where an estimated 85% of the population is at-risk [5]. The risk of malaria within the country varies dramatically as several, distinct malaria ecotypes exist. Forest malaria is a well-characterized ecotype associated with high malaria transmission due to the presence of efficient vector species throughout South and Southeast Asia [6]. In India, the forest malaria ecotype interacts with a vulnerable population to multiply the health burden. Forested areas are dominated by socioeconomically disadvantaged citizens of tribal origin who represent only 8% of the national population but 30% of reported malaria cases [7].

Since 2005 the National Vector Borne Disease Control Programme (NVBDCP) in India has introduced new interventions and undertaken several policy changes to improve malaria control [8]. ACT and LLINs achieved broad use across the nation, including many forested areas, by 2010. The objective of our study was to estimate the impact of ACT and ACT plus LLINs in a forest malaria area of India using existing cohort data. First, we estimated the effectiveness of the interventions by comparing malaria incidence before and after their introduction under routine conditions. Particularly, we were interested in whether universal coverage with LLINs and ACT was sufficient to eliminate the burden of malaria in a hyperendemic forest setting. Second, we verified reductions using indirect markers using indirect markers of malaria transmission. Third, we compared the impact of the combination of ACT plus LLINs with that of ACT alone.

## Methods

### Study Site

Sundargarh district represents ideal ecological conditions for malaria transmission with undulating plateaus crossed with rocky streams, forested hills, and paddy fields. The mean annual temperature is between 22–27°C and the average annual rainfall is between 160–200 cm. Most rainfall occurs between June and September from the ‘southwest monsoon’ and in December and January from the ‘northeast monsoon’. 51% of the district area is forested and various tribes constitute the majority (62%) of the population. Malaria transmission is seasonal with peak cases during September to January and three species of *Plasmodium* (*falciparum*, *vivax*, and *malariae*) are found here though the former is predominant. Extensive studies on vector ecology in Sundargarh district have established the role of *Anopheles fluviatilis* and *An. culicifacies* in maintaining malaria transmission [9]–[11]. The major vector is the endophagic, endophillic *An. fluviatilis* which breeds along the margins of small, slow moving streams. Its complex is comprised almost entirely by the highly efficient sibling species S with a human blood index of 0.70–0.90 and an entomological inoculation rate (EIR) of 0.23–0.39 infective bites per person per day during the high transmission season. In contrast, *An. culicifacies* is primarily zoophilic as reflected in its human blood index of 0.03 and an EIR of 0.01 infective bites per person per day.

### Study Population

We utilized data from a vaccine trial cohort which was established in Sundargarh, Odisha in 2000 and is still on-going [12]. This study retrospectively analyzed changes in malaria incidence in that cohort. We used data from 2006–2009, as our baseline, and data from 2010–2011, when interventions were present. The cohort consists of the entire population of 8 villages in deep forest areas of the district close to perennial streams. The villages fall in the catchment areas of Gurundia and Birkera primary health centres and the average distance to each is 5–10 km. The study villages are 25–60 km from the field station of the National Institute of Malaria Research (NIMR) and are accessible year-round due to their connection to all-weather roads. Previous studies of this cohort demonstrated hyper-endemic malaria including age-specific immunity [9].

### Malaria Surveillance

We conducted malaria surveillance using a trained worker who resided in each village. Village workers received training at the NIMR field station and were instructed in the preparation of thin and thick blood smears, the measurement of axillary body temperature, and the administration of antimalarial treatment. We defined a case of malaria case as any positive blood smear collected through active or passive detection among a resident of the study villages. Residents of the village reported to the worker for passive case detection in the event of any fever. In addition to passive surveillance, village workers visited every household once a week on a fixed schedule. They prepared blood smears from febrile persons or those with a history of fever. The demographic characteristics of all suspected malaria cases were obtained by interview and workers recorded the temperature at the time of screening using a digital thermometer. Slides were brought to the malaria clinic of NIMR Field Station at Rourkela where they were stained and examined by experienced technicians. We used Jaswant Singh Bhattacharya stain [13] and examined smears with a 100X oil immersion objective. At least 100 fields were examined before declaring a slide negative. Parasite density was enumerated against 200 white blood cells (WBC) and multiplied by 40 (assuming a total WBC count of 8,000 per microliter of blood). We enumerated the village population through an annual census until 2006.

### Intervention

From 2006–2009, routine vector control consisting of indoor residual spray (IRS) with DDT and synthetic pyrethroids (only one round) which was applied biannually. The use of IRS was discontinued in all villages after 2009. In January 2010, an ACT (artesunate plus sulfadoxine-pyrimethamine) replaced the use of chloroquine in all study villages for the treatment of all lab-confirmed *P. falciparum* according to the national drug policy. Treatment was provided according to age-specific dose guidelines [14]. The supply of ACT was sufficient and no stock-outs were reported. Simultaneously, in 3 study villages PermaNets 2.0, an LLIN manufactured by Vestergaard Frandsen Private Limited and procured by NVBDCP, were distributed free of cost with a ratio of 1 net per 2.5 persons to cover the entire population of these villages by the district health department as a component of their malaria control activities. Before the distribution of nets, community group meetings were organized in the villages and residents were educated on the proper and regular use of nets.

### Data Analysis

We summarized the pre and post-intervention malaria situation by tabulating standard epidemiological indicators by village. We calculated the proportion of cases due to *P. falciparum*, the annual parasite incidence per 1,000 population, and the slide positivity rate which is the proportion of blood smears positive with malaria parasites. To estimate reductions in malaria incidence we used multivariate Poisson regression among two subsets of the data (ACT villages and ACT plus LLIN villages). We modeled the number of cases as the outcome, population as the offset, year interval (2006–2009 vs. 2010–2011) as the main exposure, and adjusted for village. We examined the dispersion in our outcome and selected a negative binomial distribution to improve model fit. We used general estimating equations with an auto-regressive correlation structure to account for the clustered nature of our data within a village over time. We compared indirect markers of transmission, namely age, fever at the time of screening, and parasite density pre and post-intervention. Temperature data was missing in 234 (10.2%) and parasite density in 172 (7.5%) malaria cases. We compared age categories and proportion of cases febrile at the time of screening using the chi-squared test while we used a t-test of the log parasite density to compare the geometric means.

### Ethics

Written informed consent to participate in the cohort study was obtained from all residents. Additional consent was not obtained for each case detection and treatment encounter for malaria as these are routine health system activities for clinical care and conducted according to NVBDCP guidelines. The study was approved by the Scientific Advisory and Ethical Review committees of the National Institute of Malaria Research.

## Results

**Figure 1:**
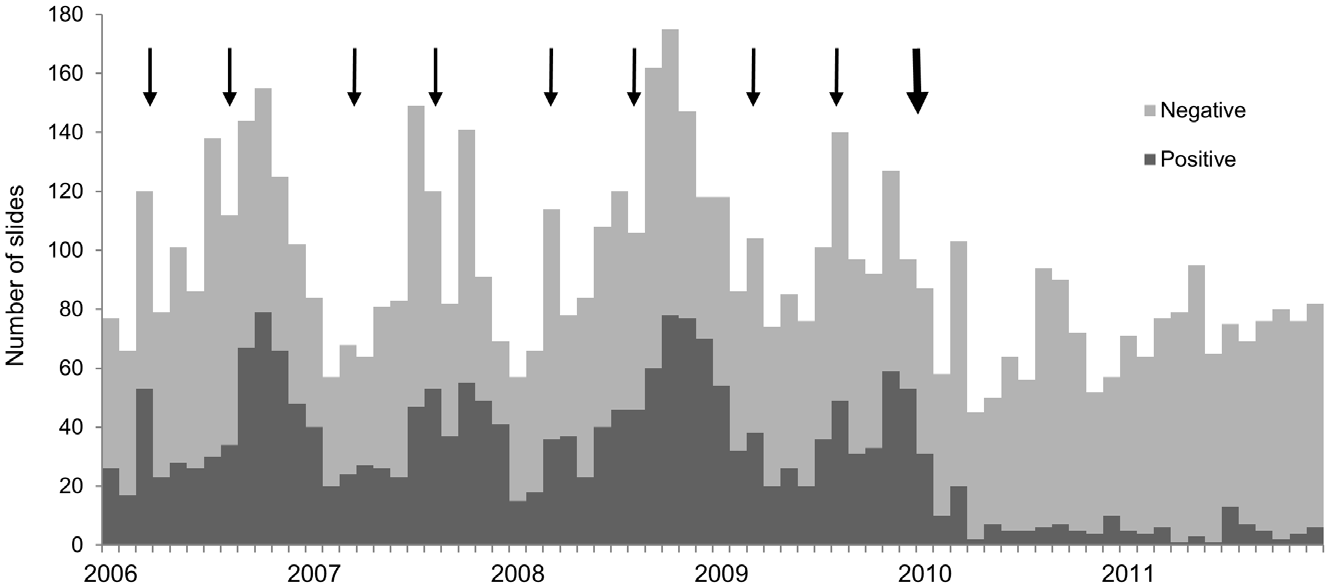
The impact of malaria control interventions (IRS - small arrows, ACT and LLINs - big arrow) on malaria cases (positive slides) and total slide collection (positive plus negative slides) by month in forest villages of Odisha, India 2006–2011.

During 2006–2009 we detected 2,116 malaria cases from 5,106 blood smear examinations (58% annual blood examination rate) in the study villages (Table 1). The burden of malaria was high with 41% of slides declared positive upon examination and most cases were due to *P. falciparum* mono-infection. *P. vivax* (n = 238), *P. malariae* (n = 11), and mixed *P. falciparum* and *P. vivax* cases (n = 9) comprised the remainder of cases. The proportion of *P. falciparum* or mixed cases with gametocytemia was 3.5% (n = 65). The mean annual incidence of malaria among the villages was 240 cases per 1,000 population. The biannual rounds of IRS appeared to have little impact on malaria incidence (Figure 1).

**Table 1:** The impact of ACT and ACT plus LLIN (shaded rows) implementation on malaria surveillance indicators in forest villages of Odisha, India 2006–2011.

		2006–2009					2010–2011				
Village	Pop	BSE	Pos	Pf%	API	SPR	BSE	Pos	Pf%	API	SPR
BK	82	214	78	86	238	36	121	7	100	43	6
BM	237	747	304	90	321	41	232	28	67	59	12
CB	85	307	131	88	385	43	142	12	92	71	8
DK	271	1118	310	86	286	28	415	23	100	42	6
RM	466	653	311	92	167	48	228	13	77	14	6
JD	156	399	213	86	341	53	136	19	68	61	14
PP	143	555	293	84	512	53	126	13	85	45	10
TM	764	1113	476	90	156	43	344	54	85	35	16
Total	2204	5106	2116	88	240	41	1744	169	85	38	10

Pop, population; BSE, blood slide examined; Pos, positive; Pf%, percent *P. falciparum*; API, annual parasite index; SPR, slide positive rate.

In 2010–2011 we detected only 169 cases of malaria in the study villages. There was a small decrease in the proportion of cases due to *P. falciparum* (85% from 88%) but this change was not statistically significant (*P value* = 0.52). *P. vivax* (n = 26), *P. malariae* (n = 1), and mixed *P. falciparum* and *P. vivax* cases (n = 1) comprised the remainder of cases. The proportion of *P. falciparum* or mixed cases with gametocytemia remained 3.5% (n = 5). The burden of malaria decreased substantially in the villages which received ACT only as well as those with ACT plus LLINs with 7% and 14% of slides declared positive upon examination. The mean annual incidence of malaria among the villages was 38 cases per 1,000 population.

In multivariate regression, the use of ACT alone in 2010–2011 decreased the incidence of malaria by 83% (IRR 0.17, 95%CI: 0.10, 0.27) compared to the same areas before the intervention when stratifying by village. Similarly, areas using ACT and LLINs decreased the incidence of malaria by 86% (IRR 0.14, 95%CI: 0.05, 0.38) compared to the same areas before the intervention when stratifying by village.

Pre-intervention in 2006–2009, 32% of malaria cases were under 5 years of age and 48% of cases were febrile at the time of screening (Table 2). With reductions in malaria transmission in 2010–2011, the mean age of malaria cases increased with 18% of cases under 5 years of age and 69% cases were febrile at the time of screening. The mean geometric parasite density of malaria cases increased in 2010–2011 compared to the pre-intervention period. Overall, 47% (n = 1076) of malaria cases were female which did not change with the intervention. There were no statistically significant differences in characteristics of malaria cases between the ACT and ACT plus LLINs villages (data not shown).

**Table 2:** Alternate measures of malaria transmission intensity before and after ACT and ACT plus LLIN implementation in forest villages of Odisha, India 2006–2011.

	2006–09	2010–11	
	n (%)	n (%)	*P value*
Age (years)			
0–4	685 (32)	30 (18)	<0.001
5–14	792 (38)	79 (47)	
≥15	639 (30)	60 (35)	
Fever (°C)			
<37.5	1011 (52)	33 (31)	<0.001
≥37.5	932 (48)	75 (69)	
Parasite count (/µL)			
Mean*	2624	3569	0.049
95%CI	2426, 2838	2711, 4699	
*geometric			

## Discussion

In 2010–2011, after ACT and ACT with LLINs were introduced, malaria incidence dramatically declined compared to the incidence during 2006–2009. Changes in indirect indicators also supported a reduction in malaria transmission. The estimated reduction in malaria incidence was not different between village with ACT alone and villages with ACT plus LLINs.

Several studies have reported decreases in transmission due to the implementation of ACT, LLINs, or combined interventions. In Zanzibar, ACT reduced malaria incidence in health facilities of one district by 71% over one year of follow-up [4]. Elsewhere, in Rwanda and Ethiopia ACT plus LLINs reduced malaria incidence in a convenience sample of health facilities by 54% and 81% respectively over one year of follow-up. The introduction of ACT plus indoor residual spraying in South Africa reduced malaria incidence in 4 health facilities of one province by 85% in the first year of follow-up and by 99% after 3 years [1]. In Thailand, ACT reduced malaria incidence in a small, randomly selected cohort by 67% and 47%, for *P. falciparum* and *P. vivax* respectively, 3 years after its introduction [2].

In our study, the implementation of ACT demonstrated comparable impact on malaria incidence up to 2 years post-intervention. Prior to the introduction of ACT, treatment failure rates for chloroquine and sulfadoxine-pyrimethamine ranged from 20–32% and 10% respectively over 28 days of follow-up among patients recruited from the referral community health centres of our study villages [15], [16]. Thus, a high impact on malaria burden due to the changeover to an efficacious treatment from a failing therapy was to be expected. However, the use of LLINs along with ACT demonstrated a modest effect on malaria incidence compared to that of ACT alone. We were surprised by this result. Model based studies suggest that in low-transmission areas a high coverage of ACT reduces transmission as much as LLINs [17]. In theory, the combination of decreased vectorial capacity and a smaller infectious reservoir should exert additive effects on malaria transmission. However, in the aforementioned studies, countries with both LLINs and ACT did not seem to experience greater declines in malaria incidence compared to countries with ACT alone although substantial differences in transmission ecotype and intervention coverage exist between examples.

Possible explanations for the observed lack of additional impact from LLINs in forest settings include the low usage of distributed nets, pyrethroid resistance in the primary vector *An. fluviatilis*, or exposure to infected bites outside the home. Based on the available evidence, we do not believe any of these possibilities are likely. Net usage measured during LLIN trials in Sundargarh demonstrated high bed net usage ranging from 70–98% in different forest villages [18], [19]. These trials also demonstrated the sensitivity of the primary vector, *An. fluviatilis*, to insecticide-treated nets with reductions in house entry rate, feeding success, person-hour density, and parity rate after intervention. Bioassays of insecticide resistance in 2009–2010 confirmed 100% mortality of the vector after exposure to pyrethroid insecticides [20]. Last, measurements of outdoor and indoor vector density documented the largely endophagic, endophillic behavior of *An. fluviatilis* sibling species S here which suggests that indoor vector control should remain effective in reducing some, if not most, exposure [9].

We were also surprised by the reduction of *P. vivax* burden, whose treatment remained unchanged (chloroquine plus primaquine), in areas receiving only ACT, though our results are consistent with the aforementioned data from Thailand [2]. Recent theories suggest that systemic febrile illness such as *P. falciparum* episodes may increase vivax malaria burden by activating a greater number of relapses [21]. Another possibility is improved treatment with ACT in mixed or misdiagnosed infections helped reduce incidence. Lower transmission would be expected to result in lower population immunity against malaria which has several effects [22]. We used indirect markers of malaria transmission to verify the robustness of our findings. We observed a shift in the age of cases towards older persons in contrast to previously observed age-specific attack rates resulting from immunity due to cumulative, frequent exposure. The proportion of patients with symptoms, in our case fever at the time of screening, also increased consistent with the theory of reduced malaria transmission and acquired immunity. Parasite density also increased in our study but this parameter is harder to interpret as expected trends are not clear [23]. Lower immunity allows parasitemia to increase more rapidly and reach higher densities early in the course of the infection. Conversely, decreased immunity could decrease the pyrogenic threshold and result in early care seeking by patients when parasite densities are lower.

Our study had several limitations. First, it is possible we missed cases if patients sought treatment from other sources. However, these villages are remote and no sources of health care, public or private, were located in the vicinity. The village surveillance worker is also well known for providing quality care for many years. Second, it is possible that confounding factors such as changes in the vector due to climate or other reasons may have driven the decreased incidence. This is unlikely as no dramatic changes in weather were reported and our post-intervention trends remained stable over 2 years. Third, we were unable to conduct a population census and update age-groups and total population size over the course of the study period. This would likely underestimate the reduction of malaria incidence in light of population growth. Fourth, although interventions were made universally available, we do not have data on their use. While we attribute the substantial remaining malaria burden to limitations in the efficacy of interventions coupled with the intensity of transmission, low net use, poor spray quality and coverage, and poor compliance with the full treatment could also explain continued transmission. Finally, unmeasured confounding or selection bias in the choice of villages could bias the results. While villages were not randomized and the number of clusters was small, both study areas had similar ecotypes and epidemiological profiles. We also sampled the entire population through passive and active case detection.

In conclusion, the introduction of ACT and ACT with LLINs dramatically reduced the incidence of malaria in hyperendemic forested, tribal villages of Odisha. In spite of universal coverage of the interventions, substantial malaria burden remained. It is unclear whether LLINs contributed substantial additional reduction in malaria incidence in the presence of effective treatment. LLINs combined with ACT may still provide additive impact in other malaria ecotypes or in forest malaria settings where the use of ACT may not be universal due to higher treatment seeking in the private and informal sectors. We recommend evaluating LLINs to determine their utility in the context of forest malaria where accessible and effective treatment is available to different extents. We also recommend the assessment of additional interventions to interrupt malaria transmission among tribal populations in forest areas.
