# Correction: The Impact of Artemisinin Combination Therapy and Long-Lasting Insecticidal Nets on Forest Malaria Incidence in Tribal Villages of India, 2006–2011

**DOI:** 10.1371/annotation/2adc258d-3eb2-4bb9-b8a9-b37ff76c6c62

**Published:** 2013-10-16

**Authors:** Naman K. Shah, Prajesh Tyagi, Surya K. Sharma

There is an error in the legend for Table 1. The complete, correct Table 1 legend is: "The impact of ACT (BK, BM, CB, DK, RM) and ACT plus LLIN (JD, PP, TM) implementation on malaria surveillance indicators in forest villages of Odisha, India 2006–2011." 

